# Towards a Better Understanding of the Sense of Safety and Security of Community-Dwelling Older Adults. The Case of the Age-Friendly City of The Hague

**DOI:** 10.3390/ijerph19073960

**Published:** 2022-03-26

**Authors:** Joost van Hoof, Jeroen Dikken, Willeke H. van Staalduinen, Suzan van der Pas, Rudy F. M. van den Hoven, Loes M. T. Hulsebosch-Janssen

**Affiliations:** 1 Research Group of Urban Ageing, Centre of Expertise Health Innovation, The Hague University of Applied Sciences, Johanna Westerdijkplein 75, 2521 EN Den Haag, The Netherlands; j.dikken@hhs.nl (J.D.); r.f.m.vandenhoven@hhs.nl (R.F.M.v.d.H.); 2 Faculty of Social Work & Education, The Hague University of Applied Sciences, Johanna Westerdijkplein 75, 2521 EN Den Haag, The Netherlands; 3 Institute of Spatial Management, Faculty of Environmental Engineering and Geodesy, Wrocław University of Environmental and Life Sciences, ul. Grunwaldzka 55, 50-357 Wrocław, Poland; 4 Faculty of Health, Nutrition & Sport, The Hague University of Applied Sciences, Johanna Westerdijkplein 75, 2521 EN Den Haag, The Netherlands; 5 AFEdemy—Academy on age-friendly environments in Europe, Krugerlaan 111A, 2806 ED Gouda, The Netherlands; willeke@afedemy.eu; 6 Faculty of Social Work & Applied Psychology, University of Applied Sciences Leiden, Zernikedreef 11, 2333 CK Leiden, The Netherlands; pas.vd.s@hsleiden.nl; 7 Department of Public Health and Primary Care, Leiden University Medical Centre, Hippocratespad 21, 2333 ZD Leiden, The Netherlands; 8 Hulsebosch Advies, Lissenvaart 43, 2724 SJ Zoetermeer, The Netherlands; hulsebosch@hm-advies.nl

**Keywords:** model, classification, ageing, qualitative, risk, hazard, risk management

## Abstract

The sense of safety and security of older people is a widely acknowledged action domain for policy and practice in age-friendly cities. Despite an extensive body of knowledge on the matter, the theory is fragmented, and a classification is lacking. Therefore, this study investigated how older people experience the sense of safety and security in an age-friendly city. A total of four focus group sessions were organised in The Hague comprising 38 older people. Based on the outcomes of the sessions, the sense of safety and security was classified into two main domains: a sense of safety and security impacted by intentional acts and negligence (for instance, burglary and violence), and a sense of safety and security impacted by non-intentional acts (for instance, incidents, making mistakes online). Both domains manifest into three separate contexts, namely the home environment, the outdoor environment and traffic and the digital environment. In the discussions with older people on these derived domains, ideas for potential improvements and priorities were also explored, which included access to information on what older people can do themselves to improve their sense of safety and security, the enforcement of rules, and continuous efforts to develop digital skills to improve safety online.

## 1. Introduction

The sense of safety and security is a theme that can be found in the earliest official documentation about age-friendly cities. The WHO’s ‘Global Age-Friendly Cities Guide’ [1] (p.1) explicitly states that:

*“An age-friendly city encourages active ageing by optimizing opportunities for health, participation and security in order to enhance quality of life as people age.”*

The WHO [1] (p.9) acknowledges that the domains of outdoor spaces and buildings, transportation and housing (as key features of a city’s psychical environment) have a profound impact on the safety and security of older people, particularly in relation to the prevention of accidents and incidents and security from crime. The WHO further mentioned the sense of safety and security in relation to the use of public transport (such as issues as theft or antisocial behaviour), which may impact the use of such services by older people. Concerning housing, homes need to be safe and situated in favourable locations. Familiar surroundings, whereby people feel part of the local community, can add to “psychological safety”. In relation to social participation, personal safety, especially during the dark hours, is considered to be a potential barrier. In relation to civic participation, there may be safety concerns at large civic events, according to the WHO report. Concerning community support and health services, the safety of the buildings is important. Social, civic and economic participation partly depend on the safety of outdoor spaces and public buildings. Promoting self-organised groups among older people for greater outdoor safety as well as providing more police are mentioned as potential solutions [1]. In the 2015 report by the WHO on the core indicators to measure the age-friendliness of cities, safety statistics are one measure to evaluate the overall progress of a city [2]. In the World Health Organization’s report [3] on future perspectives of the age-friendly movement, safety and security were, however, not mentioned.

The international scientific body of literature concerning the sense of safety and security of older people is vast, but at the same time fragmented. The physical and social quality of the living environment, including that of an age-friendly city, can impact the perception of safety and security, and these notions form the basis of Crime Prevention Through Environmental Design (CPTED). CPTED has four main principles, namely, natural surveillance, access control, territorial reinforcement and space management, which can help create a safe and secure environment for its users. The CPTED principles have been the basis of many studies on safety and security in the urban environment and how to reduce fear, reduce the incidence of crimes and improve the overall quality of life [4,5].

Despite having such design solutions implemented, Wildschut et al. [6] pointed out that the fear of victimhood among older people in the Netherlands is relatively high, while older people themselves do not run a higher risk of falling victim to a crime than younger cohorts. Concerning high-impact crimes, including threat, abuse and burglary, older people are less often victims, which is partly caused due to risk-avoidance and a different lifestyle than their younger counterparts. The subjective sense of safety of older people is also impacted by the number of social relationships in the community: the fewer contacts people have, the more unsafe they feel. A literature study from Belgium by Elchardus et al. [7] also concluded that effects of age should be interpreted based on symbolic rather than a rationalistic paradigm. Similar findings were published by Moore [8], in relation to the situation in the United Kingdom, the United States of America and Australia. In these countries, younger people run a higher risk of falling victim to a crime, not older adults. Despite these figures, American and British older people are more afraid of crime than younger people. The relationship between age and fear should not be simplified, as other factors such as sex, income, loneliness and dependency on others are of importance too.

Another theme relating to the sense of safety and security is physical safety, including incidents at home and in the neighbourhood, fires and traffic safety, as well as medication safety. Data from VeiligheidNL [9], an organisation that has been working for over 35 years to monitor all accidents in the Netherlands and develop effective programmes to encourage safe behaviour where necessary, show that in 2020, older people aged 80 years and over run the largest risks of having an incident that requires treatment at the emergency department of a hospital. The figures are up to five times higher than among younger cohorts, and are particularly higher due to fall incidents. Such incidents mainly occur at home (44%) due to tripping, falls from stairs, beds and chairs, versus another 8% of falls that occur outside on the streets. When looking at traffic accidents, older people in the Netherlands run the highest risk too. Older pedestrians run risks due to tripping, mainly because of loose street tiles and kerbs [10]. Williams [11] stated that falls, traffic accidents, burns and intoxication form the most important safety risks for older people, and these risks can be classified in terms of external and internal risk factors. External risk factors deal with environmental risks, including fires, participation in traffic and being exposed to high ambient temperatures. Internal factors relate to diminishing sensory abilities, declining mobility and numerous other forms of physical decline, as well as the intake of medication. The interaction between both internal and external factors increases the total risk for older generations. Findings from Sweden and the United Kingdom by Wennberg et al. [12] stressed the importance of the direct environment, the accessibility of the neighbourhood and a fit between the environment and person. There are numerous barriers such as high kerbs, a lack of benches, uneven surfaces, dangerous pedestrian crossings, poor illumination and the behaviours of other people in traffic which can pose risks for the safety of older people.

As a member of the Global Network for Age-friendly Cities and Communities of the World Health Organization (WHO), the municipality of The Hague in the Netherlands [13,14,15] has launched its Action Programme Age-Friendly The Hague 2020–2022 in the fourth quarter of 2020 [16]. The Action Programme calls for actions in the field of age-friendliness in the municipality. The sense of safety and security is a recurring theme in the action programme, and is linked to combatting loneliness and exclusion of older people, creating awareness for the risks older people run, such as falls and dehydration during periods of heat, overburdening of informal carers and being able to report instances of elder abuse and financial exploitation. In a recent evaluation among older people by the Municipality of The Hague [17], the sense of safety and security was an important theme in relationship to outdoor spaces and buildings, as about 20% of the respondents reported that they occasionally felt unsafe in their neighbourhood, and 2.4% that they frequently felt that way. About 3.2% of the respondents stated that they take a detour to avoid unsafe areas in their neighbourhood. The share of older people who felt unsafe in the streets during the evening hours was somewhat higher (5.7%). Even at home, about 3.4% of the older citizens did not feel safe. About 20% of the respondents did not open doors in the evening hours when the doorbell rang (compared to 12% of all citizens of the city). Still, older people in The Hague felt less unsafe in their own neighbourhood than the average citizen. When analysing areas outside of their own neighbourhood, older people felt more unsafe in areas with groups of young people (29.8%), in the city centre (23.2%) and in public transportation (22.7%), and these areas are similar to where most other citizens experience safety concerns. The share of older people who often or occasionally felt unsafe in various areas of the city was lower than the average, apart from shopping areas. In the past decade, older people are experiencing higher levels of safety in The Hague. When looking at particular subgroups, there are some differences that stand out. Older people from ethnic minority groups experience higher levels unsafety, especially in their own neighbourhood and the shopping areas. Older people with higher levels of education experienced higher levels of safety. Additionally, there were certain districts in which older people felt less safe than others, including older people living in the city centre.

Because the body of knowledge is extensive and fragmented, there is a need for an all-encompassing model or framework which describe the sense of safety and security of older people in relation to the various areas of safety and security mentioned in the literature, ranging from crime and vandalism, to physical safety in the context of one’s home, outdoor spaces and traffic, as well as the factors that influence older people’s personal sense of safety and security. Such a model or framework could help researchers to embed their specific findings in a broader perspective. Moreover, this can help policymakers to draft new policies and action plans, on the diverse safety and security themes which could help improve the age-friendliness of the municipality.

Therefore, this study aims to describe how older people experience the safety and security in an age-friendly city, what is important to them, which aspects of safety and security have the highest priority for improvement, what older people themselves do (together) in order to improve their sense of safety and security in their city and what older people need to improve regarding their sense of safety and security and the involved stakeholders. Using this information, a preliminary comprehensive classification regarding the safety and security from an older citizen’s perspective is made.

## 2. Methodology

In the following sections, the methodology of focus group sessions, the recruitment and demographics of participants and the data analysis are described in more detail.

### 2.1. Focus Groups and Setting

A sequence of four focus groups were organised, which followed the principles of a so-called “urban atelier”. In an urban atelier, both dialogue and participatory qualitative methodologies are combined [18]. Participants discuss and reflect about a certain topic, and additionally explore together what they can do themselves to solve a problem area, and what they need in order to do so.

**Figure 1:**
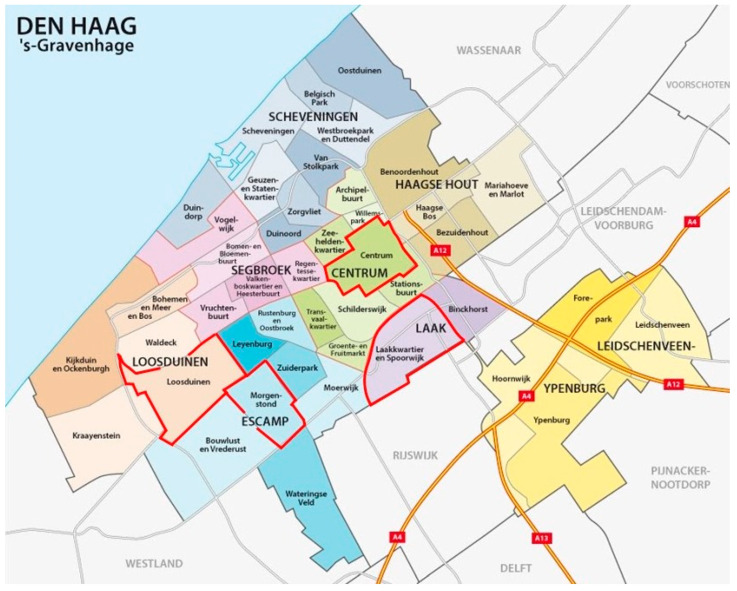
Administrative and political map of the municipality of The Hague. Neighbourhoods where focus groups were held are indicated by red borders. Taken and adapted from: Shutterstock stockvector-ID: 684913948. https://www.shutterstock.com/nl/image-vector/administrative-political-map-dutch-city-hague-684913948. (Permission obtained on 6 January 2021).

The focus groups were organised in various neighbourhoods of The Hague in order to obtain a diverse sample of older people. The sessions took place on 28 October 2021 in Loosduinen (District of Loosduinen), on 1 November 2021 in Laakkwartier en Spoorwijk (District of Laak), on 5 November in Morgenstond (District of Escamp), and on 10 November in Centrum (Centrum District) (Table 1, Figure 1). The District of Laak has a relatively young population, whereas the District of Loosduinen has a share of 28% of older people in its local population. About 95% of older people in The Hague live independently.

The focus group sessions took about two hours and were managed by a moderator and a co-moderator. The sessions followed a protocol which dealt with the research questions in a consecutive order (Appendix A). During the focus group sessions, the safety and security themes found in the literature were shown to the participants in the form of a draft framework, and the participants were asked if they had additions to the overview. Participants were asked to write down their thoughts and opinions (sometimes in duos), before sharing and discussing them with the group. Topics such as domains or areas of safety and security, personal priorities, options for improvement of the sense of safety and security and the potential for personal actions were discussed.

### 2.2. Recruitment and Demographics of Participants

Potential participants were recruited in various ways. First of all, an existing database of previous participants of age-friendly cities in The Hague received an e-mail invitation. These invitations were also shared in the network of the libraries in the city, through partners of the Knowledge Platform Age-Friendly The Hague, including a welfare organisation and the Urban Older People’s Commission, and partners of fellow initiatives in the domain of welfare, housing and care. Additional publications in local newspapers and coverage by a local radio/TV station helped to recruit participants. There were three inclusion criteria; (i) only those aged 60 (and preferably 65) years or over, (ii) who lived in their own home (i.e., not residing in institutional care), and (iii) in the municipality of The Hague, were included. A total of 38 participants joined in the focus group sessions (Table 2), of which 36 were aged 65 and over. The other two aged between 60 and 64 years of age self-identified as an ‘older person’. Education was measured with the International Standard Classification of Education (ISCED) [19]. Three levels of education were distinguished: low (ISCED 0–2), medium (ISCED 3–4) and high (ISECD 5–6). There is a slight overrepresentation of female participants in the study, of older people renting a house and of older people born in The Netherlands. As part of the demographics, participants were asked about feeling unsafe themselves. About two thirds of the participants stated that they felt unsafe at times. About one third did not experience such conditions (Table 3).

All participants signed an informed consent form. During the focus groups, which were conducted in times of rising COVID-19 cases, national requirements for group gathering had to be observed. Apart from the 38 people who participated in the sessions, not all people who signed up joined the focus groups. A small number of people (*n* = 4) did not want to join because of health concerns. Another four people who signed up for the focus groups were not able to make it due to time restraints (*n* = 3) and one due to not being able to find the room where the session was held (*n* = 1). A separate focus group that was to be held with older people from an ethnic minority group only yielded two participants, and one of these participants did not meet the inclusion criteria. This session was, therefore, excluded from the study.

### 2.3. Data Analysis

The focus groups were anonymized and transcribed verbatim. These transcripts were analysed thematically. In line with the ‘abductive analysis’ approach developed by Tavory and Timmermans [20], the analysis consisted of an iterative process of working with the empirical materials in relation to the literature on safety and security as experienced by older people. This approach included both deductive and inductive reasoning. Based on the existing literature, codes were used, such as ‘sense of safety’ and ‘prevention’, but some codes were generated inductively, for instance, regarding the different measures taken by older people, or the role that should be played by various actors in improving the sense of safety and security. During the data analysis, materials collected during the urban ateliers were studied in conjunction with the transcripts, such as notes taken during the sessions and the posters of the preliminary framework of the sense of safety and security with the annotations.

## 3. Results

**Figure 2:**
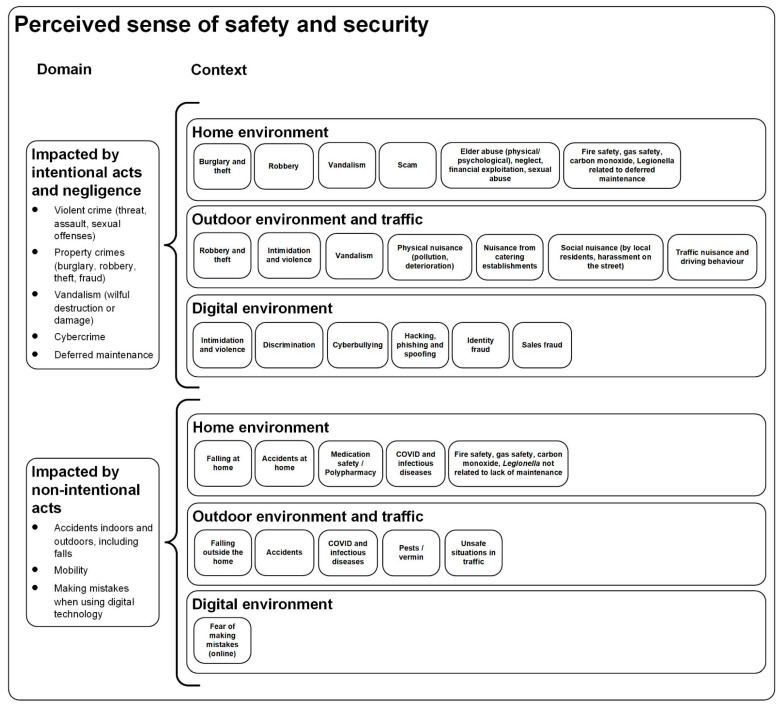
Classification of the perceived sense of safety and security.

In the Results section, we first present a classification of the experienced safety and security (Figure 2). Thereafter, we focus on the priorities in the area of safety and security, followed by answers to the question of what older people can do themselves to improve their sense of safety and security, and concluded by a set of recommendations.

### 3.1. A Classification of Experienced Safety and Security

Based on the focus groups, we made a synthesis of the aspects that contribute to the safety experienced by the participants. Within this classification (Figure 2), the experienced safety is divided into two main domains. The first domain consists of safety aspects that are influenced by intentional acts of others (doing something to someone, whether consciously or not) and negligence. The latter is particularly evident in poor maintenance of someone’s home. The second domain consists of safety aspects that are influenced by unintentional actions (something happens to someone, an accident or random unintentional act).

These two domains can be similarly subdivided into three contexts, namely the places where these security aspects manifest themselves. These contexts are characterised by concrete safety risks and issues of concern. These three contexts are their own home (the indoor space), the immediate neighbourhood or (semi) public space in the city and traffic, and the digital (online) environment related to the use of computers and technology by older people. All these contexts have their own specific set of factors that influence the perception of safety. These factors make people feel safe or unsafe. The following paragraphs describe the three contexts and factors that influence both domains.

#### 3.1.1. Safety Aspects That are Affected by Intentional Acts and Negligence

The safety aspects that are influenced by intentional acts include violent crime (threats, assault and sexual offences), property crime (burglary, robbery, theft and fraud) and cybercrime. The common thread of these aspects is that third parties who perform these acts intentionally or deliberately target older people, and that older people who are affected can only take measures to prevent these acts. Negligence (often by landlords) due to poor home maintenance also falls into this category, which can affect fire and gas safety (explosion risk), but also possible carbon monoxide poisoning. The aspects mentioned under this domain manifest themselves in three contexts.

##### Own Home

In their own home, older people may be confronted with issues such as burglary, theft (whether or not involving a con artist/scammer), robbery, vandalism, fraud and elder abuse. The latter category includes both physical and psychological abuse, with manifestations such as neglect, financial exploitation and sexual abuse. Some have already been the victim of one of these incidents, others are taking preventive measures, especially against burglary.

When we look at the factors that influence the perception of safety within this context and what makes older people feel (un)safe, many aspects were mentioned. First of all, the quality of the home (solid construction, good materials for doors and window frames, fire safety), plus a good overview from the home (view of the street and ability to see who is outside of the front door), play a positive role in increasing the feeling of security.

*“I have a new house, with a fire alarm. I still have to see what the social housing association is going to do. Carbon monoxide detectors are compulsory, but not everyone has them.“*

In addition, people mentioned the presence of preventive measures regarding burglary, such as good locks and an alarm system (or even only having the sticker of an alarm service) is recommended. A house can also be adapted, including the garden (greenery) to discourage burglars. There should be a good contact with neighbours as a preventive measure. Social control and social cohesion (in the neighbourhood and in housing complexes) can be beneficial for experienced safety (keeping an eye on things together, being vigilant together when opening/closing the entrance gate). On the other hand, older people sometimes experience nuisance from neighbours (noise pollution, fights, rubbish left in common areas).

*“There is a lot of construction going on in my street. The safety of local residents is not taken into account. Think of fine dust, construction waste, improper construction.”*

Furthermore, it is important to have a safety net in order to have someone to turn to in case of a life event or an emergency, including one’s general practitioner. At the same time, being able to maintain a social life with relevant others may become difficult, for instance, due to people passing away. Therefore, it is important to keep making a conscious effort to establish new contacts, which requires resilience and an active attitude.

##### The Neighbourhood and Traffic

In their neighbourhood and in traffic, older people can be confronted with issues such as robbery and theft, threats and violence. These things may happen on the street and are a source of concern. In addition, there is vandalism, nuisance from cafés or nightlife establishments and physical nuisance (including pollution and decay) that people may experience. In the social realm, there are social nuisances caused by local residents or all kinds of forms of harassment on the street (liability/fire) are a source of concern.

*“What I find frustrating is that there are a lot of cleaning vans driving around but they don’t do anything. [...] That’s not what they are there for. The municipality has all of its departments. Everyone has their own task, but they don’t respond to anything they see.”*

People also experience all kinds of traffic nuisance, partly due to the driving behaviour of others. There are concerns about aggressive behaviour and other misbehaviour in traffic, including parking violations on sidewalks. These parking violations are not only a problem caused by motorists, but also by bicycles, mobility scooters and others. Respect for public space is also important for the sense of safety. Participants experience an increasing lack of respect and friendliness on the street, in traffic and in public transportation.

*“Scooters and e-bikes drive too fast, especially on roundabouts.”*

*“Give older people compulsory mobility scooter training.”*

If we look at the factors that influence the experience of safety within this context, and what makes older people feel (un)safe, the following issues came to the fore. First, there is the neighbourhood reputation, which influences the safety experience. Social cohesion in the neighbourhood, social networks and the way people interact in the neighbourhood can make the difference.

*“I have known the young people here since they were children. They know me. I can call them to account for their behaviour if necessary.”*

The overall quality, design and maintenance of the public space play a role in the experienced safety of older people. Aspects such as good street lighting and having a good overview of the space play a role in this. Physical degradation (vacancies, pollution, overdue maintenance, dilapidated street furniture) reduces the sense of security. Public drunkenness and drug use in the street, loitering youths and unleashed dogs have a negative impact on the safety experience. Older people consciously adapt their behaviour. For example, some older people do not take a handbag to the market, do not go out at night and pay close attention when using their bank card (and security code) outside on the street or in another building. There is a need for the presence, visibility and accessibility of formal and informal surveillance, including by police. This often leaves much to be desired.

*“There are aggressive beggars in our neighbourhood. The police know that. It has been discussed in the city council, but they say there is no record by the police. The police do not enforce.”*

##### Digital Environment

In the digital environment, issues such as threats and violence, discrimination and cyberbullying are mentioned. In addition, there are issues that have more to do with scams, such as hacking, phishing and spoofing, identity fraud and sales fraud. If we look at the factors that influence the experience of safety within this context, and what makes older people feel (un)safe, the following elements were brought up in the focus groups. There are widespread concerns about the accessibility, usability and convenience of services and facilities. Especially if these were once offered physically and are now available in digital format. There is a broad need to provide physical alternatives to digital services.

*“The municipality reduces security by digitising everything.”*

There is a need for better information services, with information not only available digitally, but also in person, for example in community centres or at the municipality.

*“There will be digital government information points in libraries. But what if people cannot physically go there?”*

Older people need to acquire digital skills if they do not possess these yet. Few older people are aware of the possibilities for learning digital skills or the possibilities for using technology in daily life, and where they can find information about this.

Many also find communication with (semi) government agencies via the Internet difficult and unsafe (for example: filing a tax return, health insurance, arranging municipal affairs). There is an increasing number of misleading e-mails, phone calls, and so on. Phishing is a major issue in perceived insecurity.

*“Really old people, who cannot use digital resources. They literally have nowhere to turn, they are vulnerable to scams.”*

Children (if present) are often used to assist in digital activities, especially when doing online tasks such as banking, filling in forms and making online purchases (important during COVID-19). Older people do not want to be dependent, not even on their children.

*“Older people become isolated, especially if you don’t have children or your children live far away. Not everyone has a helper who can be trusted.”*

#### 3.1.2. Safety Aspects Affected by Unintentional acts

The domain of safety aspects that are influenced by unintentional acts includes things such as accidents in and outside the home, including falls, mobility issues and making mistakes when using digital technology. The common thread in these aspects is that older people through misfortune or unintentional actions experience something that affects their safety. The aspects mentioned under this domain manifest themselves in three contexts: the own home, the neighbourhood and traffic and the digital environment.

##### Own Home

In their own homes, older people may face issues such as falls and accidents/incidents in the home, as well as medication-related accidents (polypharmacy), when medication is taken incorrectly. In addition, there are issues such as fire safety and carbon monoxide poisoning at home that are not caused by negligence and poor maintenance.

If we look at the factors that influence the experience of safety within this context, and what makes older people feel (un)safe, the following issues were mentioned. First of all, the physical quality of the home is important, such as if there are doorsteps. These can be dangerous and cause falls. One may also take a critical look at the home furnishings (such as removing mats, rugs, electrical cords, and so on) and look at maintenance of the home. Installing fire and smoke detectors, both in their own home and at their neighbours.

*“I hope that the municipality can make adjustments in the house. There is also potential for abuse. There should be appropriate information about it.”*

Supervising medication intake and the dispensing of medication is seen as an important factor to promote medication safety. The presence of alarms and signalling systems, as well as access to a call tree (cascade calling) are seen as positive in terms of experienced safety. One’s own physical and mental state are seen as protective factors and contribute to the experience of safety.

*“Make sure you stay fit, keep your balance, remain physically strong. Take the initiative yourself, greet people, approach people actively, in a positive way.”*

*“Social life changes when you are scared. How do you gain more self-confidence? Challenge yourself? But what do you do if you are faced with three people? It doesn’t matter how strong you are.”*

Finally, people should set up a neighbourhood prevention team as a preventive measure to increase the feeling of safety.

##### Neighbourhood and Traffic

In the neighbourhood and in traffic, older people can be confronted with things such as falling outside the home (including on the street), other accidents and mishaps, vermin and traffic (unsafe) situations. If we look at the factors that influence the experience of safety within this context, and what makes older people feel (un)safe, the following issues came to the fore.

The overall design and maintenance of public spaces and public greenery affect the experienced safety, such as the quality of the pavement and any loose street tiles. A well-maintained and clean street enhances the sense of safety and security.

*“The sloping transitions to the sidewalk. Well intended, but if you don’t see these, you bump against them and fall.”*

Good lighting is important for experienced safety. People do not like dark streets, alleys and corners.

*“I suffer from vertigo, if the street is not level, the street is dangerous for me, especially at night in the dark.”*

The design and construction of infrastructure must take into account aspects of safety and the behaviour of others in traffic. Obscure and unsafe situations in traffic can lead to a general feeling of unsafety in traffic. This includes paying attention to correctly parked vehicles, including cars and electric (share) vehicles that are parked on sidewalks and thus obstruct passage. One’s own driving ability and behaviour are also mentioned as limiting factors.

*“The mix of pedestrians, cyclists, mobility scooters, electric bikes, cargo bikes, all mixed up together, going at different speeds, and you don’t always hear them coming.”*

*“As you get older, you sometimes don’t see as well and it’s harder to oversee situations.”*

##### Digital Environment

In the digital environment, people mention issues that have to do with the fear of making mistakes in the online environment and, related to this, being very careful when performing digital actions.

There are widespread concerns about the accessibility, user-friendliness and convenience of services and facilities, particularly if these were offered in-person before and are now provided in digital format. There is a broad need to have physical alternatives to digital services. There is a need for better information services, with information not only being available in a digital format. Older people need to acquire digital skills if they do not have these yet sufficiently. There is insecurity at work due to reduced productivity, having to care for older parents, or not being able to keep up with digitalisation.

*“There are many older people (especially those who are less digital savvy) who have a problem because they lack information. There are no neighbourhood newspapers, nor is there an accessible map of what is going on in the neighbourhood.”*

### 3.2. Priorities in the Area of Safety and Security

Older people do not often feel they are the victim of so-called ’high-impact crimes’, but they do fear, for instance, a burglary. They arm themselves against burglary (alarms, reinforced hinges and locks, keeping an overview) and feel that the municipality and housing corporations should facilitate them in this.

Older people consider an area-oriented approach, with a focus on specific problems in neighbourhoods and on the streets, a priority. They can specifically point out neighbourhoods and streets where there is an accumulation of problems (rubbish on the street, poor maintenance, vermin, people begging, nuisance, unsafe [traffic] situations). Older people also consider it a priority that they are involved in the analysis and solutions of the problems (for example by participating in a joint neighbourhood walk).

Older people usually see solutions in a combination with law enforcement, taking action, concrete interventions and improving communication (an exchange of norms and values) with and between residents. However, many older people feel that they have no influence on the actions of the municipality and other institutions, for example in the area of traffic improvements (extending marked pedestrian crossings to the cycle path or separating fast and slow traffic) and adjustments in public spaces (sidewalks, lighting). Not being involved in changes (for instance, digitalisation) gives them a sense of insecurity, of not being acknowledged. This can lead to accidents (falls, digital ‘accidents’). The priority is therefore to involve older people in finding solutions.

Finally, many older people are afraid of falling and other accidents (including digital), sometimes due to reduced sight or hearing. It is a priority for older people to be acknowledged and receive attention.

When summarising the priorities, participants wished to see a change in the reckless behaviour of people in traffic, and a consideration of the needs of older people taking part in traffic. Such needs also include a better division of pedestrians and cyclists moving around where their paths cross. There is also a need for improved illumination on the street and the repairing of uneven pavements. Second, people wish to see solutions for safety and security in the digital environment, as they feel being excluded and dependent on the help of others. Older people are very aware of their vulnerability due to both physical decline and cognitive challenges that arise from ageing and the progress of society. They ask policymakers to take these challenges seriously, and to explore and co-produce solutions together. Such solutions contain both hard and soft controls, such as enforcement and better communication and options for older people in terms of training and information on safety and security.

### 3.3. What Can Be Done to Improve the Sense of Safety and Security?

When it comes to increasing the feeling of safety and security in the city, there are things that older people can do themselves and together with others. It may also be the case that they cannot do it alone and need help from those around them (and others) to increase their sense of safety.

#### 3.3.1. What Can Older People Do Themselves and Together to Increase Their Sense of Security in the City?

When we look at what older people can do themselves and together with others to increase their sense of security, a number of solutions are mentioned. Reporting events and situations is among the solutions mentioned, but follow-up seems to be essential.

*“I’m part of the neighbourhood intervention team, for example, we report which lampposts are broken. We always walk in pairs, a man and a woman. Sometimes together with enforcement officers.”*

*“I get annoyed when people ask us if we have solutions for what the government can do when the same government is taking away security by digitising everything.”*

*“Most people in this group know the service apps and numbers, but some have experienced that the municipality doesn’t follow up on their reports.”*

People stress the importance of having a good social life and the need to ensure good social contacts. People should take the initiative, greet people and approach people actively in a positive way. The support from children, who can help with certain things (such as the Internet, and some children will have the key to the participants’ home), is valued. Older people should have trust in themselves (attitude) and live more consciously.

*“Being older is a wonderful time in your life, if you can adapt yourself and if your environment adapts as well. Be considerate of each other.”*

In addition to these social and psychological aspects of older people themselves, a number of physical aspects were also mentioned, such as maintaining physical fitness: keeping fit, keeping your balance, being physically strong. Adapting to what one can still do, but also trying to change things, were mentioned. Participants also mentioned examples of preventive and avoidant behaviour.

*“I don’t cycle anymore because I don’t trust myself.”*

On the one hand, these focus on the home, such as organising your home to reduce the risk of falling: make sure there are fewer obstacles in the house (cords, rugs), support in showers and toilets. Preventive measures include covering the stairs with carpets, non-slip floors in the bathroom and handrails, avoiding using stairs or ladders, using emergency response systems, changing from gas to induction cooking and having the social housing association install a smoke detector or a carbon monoxide detector.

*“Make sure the house is well secured, so you can sleep well when your children are out of the house.”*

On the other hand, some examples focused on the neighbourhood and traffic, such as not going out at night and not opening the door at night to anyone, were mentioned. An example that was mentioned here was agreeing on a signal with acquaintances (such as calling three times) or giving the key to family members. Another example was putting stickers on the door saying, ‘*I don’t open the door just for anyone*’. Other people stated avoiding traffic at certain times and going for grocery shopping during quiet times. In order to prevent crime, people should pay attention when they go out, for example by leaving their handbag at home when they go to the market.

#### 3.3.2. What and Who Do Older People Need (More of) to Increase Their Sense of Security in the City?

If we look at what and who older people need to increase their sense of security in the city, the following things are mentioned when it comes to their own home. Some older people wish for support in the shower and toilet, and seek funding for this. Others wish to install a peephole in the door or a camera so you can see who is outside your door, install a smoke detector and carbon monoxide through social housing associations. Other examples concerned the neighbourhood and traffic, such as making the inner city more accessible for pedestrians; providing alternative routes for cyclists, clearly separated from pedestrians; extending crossing times; improving the maintenance of pavements, provide clean public spaces and better lighting. Others suggested closing (semi-)public areas after certain hours, to provide better supervision, more enforcement by police and camera surveillance, and to increase the visibility of neighbourhood police.

*“Why not create a closed city centre, accessible only to pedestrians. There are good examples of this in other cities.”*

*“Be more considerate of older people when designing and structuring the city.”*

A number of solutions to crime were also specifically mentioned. Several participants have dealt directly or indirectly with burglary and theft. Participants mentioned some of the following examples, such as installing an alarm system, locking the door when coming home and installing a motion-detector lamp that turns on when it detects movement outside the home. A number of participants gave examples of the need for more information on issues such as digital security, elder abuse (including financial abuse), living wills, adaptations in the home and modern technology. The importance of raising awareness about older people in society was also mentioned, especially among young adults, and not just older people as ’objects of concern’.

*“Involving parents of loitering youth in solving the nuisance problem.”*

Finally, older people wish to create solidarity in the neighbourhood:

*“Getting to know the people who are there, we have already held coffee mornings twice a week for local residents. Not everyone who is homeless is an addict or a nuisance.”*

### 3.4. Recommendations

The recommendations and conclusions focus on what the municipality can do to improve or support the sense of safety and security for older people. Many of the possible solutions are in the domain of what older people can do themselves, the housing corporations or of the national government. The municipality can put a number of domains on the agenda in the so-called ‘safety triangle’ (the consultative body in The Netherlands consisting of the mayor, the district chief of the police and the prosecutor), in performance agreements with housing corporations and in subsidy agreements with welfare organisations and affiliated institutions. Some participants referred to themselves: make sure you stay physically and mentally fit. Make sure you have good security and good locks. Others ask the municipality to facilitate better security, for instance, through better enforcement (clean streets, but also traffic behaviour); education and information (on reporting crime and rules of conduct); repairing streets and sidewalks (overgrown roots, uneven pavement after cable repair, clearly marked kerbs) as well as street lighting; cleaning streets (including around shops and businesses); and promoting contact among residents by exchanging standards and stimulating mutual understanding.

#### 3.4.1. Own Home

Many older people are not aware of what they can do themselves to make their home safer. Both in terms of burglary protection and safety, but also in preventing falls, and so on. Education and information are needed about how to report a crime, tools and resources (also technological) in and around the house and how to recognise scams/cons. Enforcement is required in case of renovations in a house and the impact on neighbours.

*“Why doesn’t the municipality issue a welcome booklet for residents? This has been cut.”*

#### 3.4.2. Neighbourhood and Traffic

The longer people live in a neighbourhood, the safer they feel. Especially if they are active in the neighbourhood and have seen today’s young people grow up and feel they sort of know them. When there is a group of newcomers (migrant workers, homeless people, etc.), older people find it important to get to know them and also that the newcomers follow the regular norms of the neighbourhood. Enforcement and communication are both issues that the municipality could address. Furthermore, there should be more education and information about the municipal reporting app.

*“A neighbourhood walk can help clarify things. A walk with the social housing association, police and the municipality.”*

There is also concern about the quality of the pavement as well as about roots of trees that grow under the bicycle paths, pushing up the tiles and asphalt.

*“When the streets are fixed, they start laying cables again and it’s the companies that lay the cables who even out the pavement, not the professional road workers.”*

*“Are the tree roots guided? This is possible, right? It’s also expensive to keep redoing the bicycle path.”*

#### 3.4.3. Digital Environment

Older people are concerned and angry that they are becoming too dependent on their children or others to be able to participate. Digital skills should be offered much more and be customised, accessible and applicable to everyday life. The latter is especially important: the actual ability to understand and learn the daily current skills needed, for example DigiD (the Dutch national digital identification code for citizens), QR codes, voice-activated messages and websites, ’my environments’ for institutions, filing a report or tax return, using municipal apps and settings and use of mobile telephones.

*“If you are not digitally literate or don’t have a DigiD, you can’t force people to do everything digitally, but then I think they should also offer people help to implement this.”*

The websites of municipal and subsidised organisations should be clear and voice-activated and have a ‘read aloud’ function, preferably in several languages. Attention should be paid to people with low literacy skills. Older people sometimes let their care workers do their shopping with their debit card. The temptation of financial abuse is present here. There should be more attention on this issue or to provide solutions, so older people feel safe. Finally, there is a growing need for information on issues such as so-called ‘living wills’.

*“I have also noticed that when I go to a digital information point for older people, I am completely outdated. So, I go and ask for help with appliances, whether it’s a camera or something else. I don’t get that [information] anymore, because I should have asked for that some 20 years ago.”*

## 4. Discussion and Conclusion

The current findings have been captured in a comprehensive classification concerning two domains and three distinct contexts. This classification shows an extensive set of elements that constitute this phenomenon, many of which had already been identified in the existing literature. Such elements concern falls and incidents, being safe from burglary and theft, as well as being safe in traffic. Cybercrimes are an emerging theme that came to the fore in the various focus groups too. To date, these risks were often classified in terms of internal and external causes. The present classification makes a clearer distinction between safety aspects that are influenced by intentional acts of others and negligence on the one hand, and safety aspects that are influenced by unintentional actions, which manifest in the contexts of the person’s own home, the immediate neighbourhood and traffic, and the digital environment. Many of these safety and security-related topics of the new classification have been studied as separate elements, and the existing body of knowledge contributes to its validity. Due to the large variety of topics related to the sense of safety and security, studies often remain unconnected from each other, although they all contribute to the perceived safety and security of older people. The new classification helps structure and bring these studies together within a larger ecosystem of safety and security, as is deemed important by older people themselves. Apart from structuring the body of knowledge, gaps in the literature and in practice can also be identified, and such gaps may deserve further study and attention in the future. The classification may help researchers and policymakers to have a better understanding of the full width and scope of the theme, instead of focussing on separate elements or specific contexts. The present study shows that policymakers have many parameters at their disposal which can be controlled, steered and improved in order to increase the sense of safety and security.

The sense of safety and security is an integral aspect of the age-friendly agenda of the WHO [1], in particular in the domains of outdoor spaces and buildings, transportation and housing. This study further adds to the understanding of what safety and security entail for older people living in an age-friendly city, as there is a vast but fragmented body of knowledge, which has not been reviewed and classified from the perspective of older people themselves. When looking at the findings, it is interesting to see how all of this fits in the context of age-friendly cities in terms of policy and practice. De Donder et al. [21] raised a number of issues for age-friendly policy and practice. The researchers stressed that architects, environmental planners, urban developers and geographers could base future work on the notion that the physical quality of the local environment can contribute to higher feelings of safety, and that, therefore, environmental design could be encouraged. These notions also reflect the work laid down in the CPTED-related guidelines [22,23]. De Donder et al. [21] further stated that intervention programmes should be aimed at older people feeling at home and safe in their neighborhood. Such programmes could also improve the quality of life of the community.

The current study has shown that the sense of safety and security, and solutions to related challenges, encompass more than simple and more intricate environmental design aspects. Participants came up with a long set of recommendations about how to improve the safety and security situation in their lives, both in terms of what they can do themselves, and what they need from other stakeholders such as the municipality. This also came to the fore in a study by Cecatto and Bamzar [24], who pointed to various factors that impact the feelings of safety and security among older people, including (1) personal factors of declining health and of physical vulnerability, as well as one’s financial situation; and (2) the quality of the living environment (including public lighting, overall maintenance levels, graffiti) and its social networks and cohesion, including feelings of loss of control due to changing demographics. Similar elements can also be found in studies by Buffel et al. [25] and Buffel and Phillipson [26]. De Donder et al. [21] examined the impact of the perceived design of the neighbourhood on feelings of unsafety in later life. Their study found that a neighbourhood which is perceived to be physically adapted in terms of accessibility and distance to services heightens feelings of safety. During the focus group sessions, many participants raised concerns about not being given a voice by the municipality in broader developments that took place in the living environment, and which impacted the quality of the living environment and social cohesion. A Technical Report by the European Committee for Standardization [22] contains a roadmap for engaging with a wide range of stakeholders, including residents and local authorities, on different scales of the urban fabric (street, neighbourhood, city levels). The roadmap is one of the instruments for municipalities to explore the solutions for safety-related challenges with older people.

The classification that was made as an outcome of this study shows that the aspects that are influenced by intentional acts of others and negligence require a different approach than safety aspects that are influenced by unintentional actions. Both solutions and types of stakeholders involved differ for both domains, and for the three contexts of the person’s own home, the immediate neighbourhood and traffic, and the digital (online) environment. By focussing only on the environmental design solutions, the scope of potential improvements to the sense of safety and security would be too narrow and would not cover all problem areas.

During the focus group sessions, some elements that do appear in the literature were not raised by the participants. The elements include aspects of being able to work in safety and security as a community nurse when providing care to older people, and the safety of assistive technologies and equipment such nurses have to work with. There was no mention of the safety of care technology from the point of view of informal care, nor from the perspective of older users themselves. A sense of safety and security in a care relationship was also not mentioned, nor was data security concerning medical data, and the safety of food and meals were not discussed either. There is a large body of knowledge relating to the sense of safety and security in the domain of home care [27,28,29,30,31,32,33]. One of the reasons for the omissions is the fact that the scientific literature is often written from the perspective of professionals and organisations and does not necessarily reflect the outcomes of the subjective perception of older people. The topic of elder abuse was also mentioned in all of its facets, but not in great depth. One could ask oneself the question of if this topic is suitable for discussing in a plenary meeting, such as in a focus group session.

As this study looked at the sense of safety and security of older people, it remains a question if older people run higher risks of being victims than other age cohorts. When looking at the experiences of older people with crime and nuisances, several Dutch organisations including KBO-PCOB [34,35], the Centre for Crime Prevention [36] and the Ministry of Justice and Security [37] have launched campaigns in recent years, focusing on raising awareness and the provision of information concerning the theme of older people and safety. These campaigns spanned a wide range of elements, including burglary and scams, Internet fraud and safety on the streets. Statistics Netherlands (CBS) [38] made a distinction between four types of crime, including violent crimes, property crimes, vandalism and cybercrimes, and concluded that the sense of victimhood was higher among cohorts aged between 15 and 44 years old, than people aged 65 years and over. Actual figures about falling victim to a crime showed indeed that people aged 15 to 24 years old (17%) and people aged 25 to 44 years old (16%) were twice as often victim of a crime compared to people aged 65 and over (8%), in particular when looking at violent and property crimes. Older people are less often victims of violent and property crimes and of vandalism in the Netherlands than their younger counterparts. This is even true for cybercrimes (sales fraud and hacking in particular). Only identity fraud is experienced more often by people aged 65 years and over. The Netherlands Institute for Social Research (SCP) [39] stated that people in The Netherlands have developed an improved sense of safety in their surroundings over the years, and also showed that younger cohorts are more often victims of a crime. All crime rates are decreasing, apart from cybercrime. In 2019, people aged between 15 and 24 years old were victims of property crimes and cybercrimes 2.5 times as often as people aged 65 years and over. These are somewhat different figures than those by Statistics Netherlands. Although concerns among older people are genuine, policymakers need to weigh them against the concerns of other cohorts. In addition, the participants of the focus group sessions had different views of safety and security than the outcomes of the so-called Safety Plan 2019–2022 of the Municipality of The Hague [40,41]. For this plan, 3286 citizens of The Hague were consulted through an online questionnaire. Again, the overall sense of safety and security has improved over the last decade. There are three domains in which the municipality takes actions against crimes. First, actions against high-impact crimes; second, a problem-oriented approach in districts and neighborhoods; and third, actions against undermining (both through criminal law and administration). The accents of this third pillar include strengthening the city’s resilience, both against radicalisation and polarisation as well as terrorism and cybercrime, actions against domestic abuse and undermining, and preventive measures against people with mental issues. Older people in the focus groups worry about cybercrime and overlook areas as undermining, polarisation and radicalisation, which seem to be distant and unknown concerns.

New guidelines for age-friendly cities and communities may need to be developed, including instruments to measure the perception of safety and security by older people, in order to move the age-friendly agenda forward. Therefore, additional parameters and core indicators may need to be identified, in line with other gaps in the age-friendly agenda that have come to the fore [42,43,44,45,46,47,48]. Such instruments, parameters and core indicators could then be used, as rightfully stated by De Donder et al. [21], to create intervention programmes that are context-specific and have local embedding, which reflect local circumstance and build on the knowledge and experiences of the local residents. As age-friendly environments target people of all ages, the outcomes of this study could also be of relevance to younger cohorts, who, according to the national statistics, are more at risk of falling victim to crimes than older people. In addition, there may be a need for quantitative methods for evaluating and assessing the sense of safety and security among older citizens. De Donder et al. [21] pointed to a questionnaire developed by Elchardus and Smits [49], which measures general feelings of safety, and which has been adapted for older people. This questionnaire, coined Elders Feelings of Unsafety (EFU) scale, contains eight items that relate to going out on the streets at night, safety outdoors, opening doors when the bell rings and home alarm systems. Given the limited scope of the EFU scale and the wider implications of our new classification, there is room for a new quantitative questionnaire focusing on the wider theme of safety and security as experienced by older people. This requires a procedure that needs to be performed in a rigorous and transparent manner, for which the outcomes of the current study may provide a good first step in defining the construct that is to be measured. Ideally, making a new questionnaire should follow a step-by-step approach in line with the criteria stated by the COnsensus-based Standards for selection of health Measurement INstruments (COSMIN) [50,51]. The COSMIN initiative aims to reach a consensus about which measurement properties are considered to be important, their most adequate terms and definitions, and how they should be assessed in terms of study design and statistics. Given the current state-of-the-art in the literature concerning the sense of safety and security, we believe that further research is needed to validate the current proposed classification before being able to develop an all-encompassing questionnaire on this phenomenon.

The current study has shown that the sense of safety and security is a complex and multi-faceted phenomenon. It can be classified into two main domains: a sense of safety and security impacted by intentional acts and negligence, and a sense of safety and security impacted by non-intentional acts. Both domains manifest into three separate contexts, namely the home environment, the outdoor environment and traffic and the digital environment. More research is necessary to complement this classification and develop and validate it further into a complete framework or even (predictive) model and additional questionnaire. In the future, correlations and interactions between elements of the classification may be explained and used for the design and development of interventions and their implementation. One point of attention for future research could be the subtle differences between the experienced safety and security of age cohorts, people living in various neighbourhoods and with different cultural or ethnic backgrounds. Nevertheless, based on the review of the literature, themes seem be more universal in nature than merely valid for the situation encountered in the four neighbourhoods of The Hague. There is room for further additions and clarifications of the proposed classification. Policymakers could use the results of the current study, as well as the classification, to assess current policies and evaluate the effects of action programmes, and help steer and tune future actions and agendas by a wide range of professional stakeholders including the municipality and the police. The current study and its results may also help the municipality to identify gaps in the current policies, for which future interventions are required.

This study helps to structure existing research findings within a classification, and provide an overview of the place and relevance of existing studies in relation to other topics in the domain. Moreover, age-friendly agendas of global network members should explicitly address the safety and security needs of older people and draft a mutual agenda on how to improve aspects that impact this perception, ideally through a shared action plan that requires the input and effort of both older people and municipalities alike.

## Figures and Tables

**Table 1:** Population of The Hague and its districts (on 1 January 2021) according to DHIC/Gemeente Den Haag/Dienst Publieke Zaken (https://denhaag.incijfers.nl/jive) (accessed on 23 January 2022).

Area + District	Total Number of People	Percentage of People aged 65 Years and Over [%]	Total Number of People Aged 65 Years and Over
Loosduinen (Loosduinen)	50,211	28%	14,064
Laakkwartier en Spoorwijk (Laak)	46,443	7.9%	3686
Morgenstond (Escamp)	128,808	12.7%	16,393
Centrum (Centrum)	105,996	11.7%	12,405
Municipality of The Hague (Total)	549,163	14.8%	81,410

**Table 2:** Demographic data of the participants (*n* = 38).

Sex	Male	*n* = 9 (23.7%)
	Female	*n* = 29 (76.3%)
Age	Mean (SD)	74.3 (6.6)
Country of birth	The Netherlands	*n* = 32 (84.2%)
	Suriname/former Netherlands Antilles	*n* = 5 (13.2%)
	Guyana	*n* = 1 (2.6%)
Educational level	ISCED 0–2	*n*= 9 (23.7%)
	ISCED 3–4	*n*= 27 (71.0%)
	ISCED 5–6	*n*= 2 (5.3%)
Years of living in The Hague	Mean (SD)	54.2 (21.0)
Type of dwelling	Owner-occupant	*n* = 13 (34.3%)
	Social housing	*n* = 17 (44.7%)
	Private rent	*n* = 7 (18.4%)
	Missing value	*n* = 1 (2.6%)
Living together with a spouse or partner (%)		*n* = 12 (31.6%)
Receiving care (%)		*n* = 13 (34.2%)
Living with one or more chronic conditions (%)		*n* = 19 (50%)
Using a wheeled walker or wheelchair (%)		*n* = 7 (18.4%)
Rating quality of life on scale 1–10 (SD)		7.8 (1.9)

**Table 3:** Answers to the question: Do you ever feel unsafe?

Area + District	Yes	No	Does Not Know, Not Answered	Total
Loosduinen (Loosduinen)	6	8	0	14
Laakkwartier en Spoorwijk (Laak)	6	2	0	8
Morgenstond (Escamp)	8	2	0	10
Centrum (Centrum)	5	1	0	6
Total	25	13	0	38

## Data Availability

The data presented in this study are available on request from the corresponding author.

## Appendix A. Protocol for focus group sessions

The questions posed to the participants were:

1. What is important to older people themselves in terms of their sense of safety and security? (Write down thoughts on a Post-it^®^ note, to be collected by moderator).
2. Which aspects of safety and security have the highest priority for improvement? (Dialogue and writing down comments on a flipchart).
3. Do you ever feel unsafe? (Using a flowchart).
4. Showing a concept classification of the sense of safety and security, as ask participants if there are elements which are important to them but which were not discussed before? (Deepening the themes through dialogue).
5. What can older people themselves do (together) in order to improve their sense of safety and security in their city? (Dialogue in duos, plus plenary discussion using a flipchart)
6. What do older people need to improve their sense of safety and security, and who are the stakeholders involved? (Write down thoughts on a Post-it^®^ note, to be collected by moderator).
7. Final concluding question if there are any other thoughts or remarks that need to the shared.
